# Supplementary material for: Deep learning-based enhancement of epigenomics data with AtacWorks
Source: Nat Commun. 2021 Mar 8;12:1507. doi: 10.1038/s41467-021-21765-5 (PMC7940635; doi:10.1038/s41467-021-21765-5)
Supplement: Supplementary file 1 — Supplementary Information [file 41467_2021_21765_MOESM1_ESM.pdf]

Supplementary Information

Deep learning-based enhancement of epigenomics data with AtacWorks

Avantika Lal[1,*], Zachary D. Chiang[2,*], Nikolai Yakovenko[1], Fabiana M. Duarte[2], Johnny Israeli[1 †], Jason D. Buenrostro[2 †]

[1]NVIDIA Corporation, 2788 San Tomas Expy, Santa Clara, California 95051, USA.
[2]Department of Stem Cell and Regenerative Biology, Harvard University, Cambridge, Massachusetts 02138, USA.
*Equal Contributions
† Co-corresponding

Correspondence should be addressed to: jisraeli@nvidia.com or jason_buenrostro@harvard.edu.

This document contains:

Supplementary Note 1

Supplementary Tables 1-14

Supplementary Figures 1-9

Supplementary References

Supplementary Note 1

Another study[1] recently reported the use of a deep learning model for denoising and peak calling from low-input ATAC-seq. The software used in this study was released as PillowNet (https://github.com/daquang/PillowNet).

Here we evaluate and compare:
1. The AtacWorks and PillowNet software tools;
2. The ResNet architecture used in AtacWorks with the U-Net architecture used in PillowNet; and
3. The ResNet architecture used in AtacWorks with an independently designed and tuned U-Net architecture.

1. Comparison of AtacWorks and PillowNet softwares

A bulk ATAC-Seq dataset from CD4+ T cells was sampled to a depth of 50 million reads to generate clean, high-coverage data, and then subsampled to a depth of 1 million reads.

Both AtacWorks and PillowNet models were trained, with their respective default parameters, to learn a mapping between the noisy and clean datasets. The code at https://github.com/daquang/PillowNet was used to train the PillowNet model. Only a 12 Mb region of chromosome 1 was used for training and models were trained for 25 epochs.

Since the U-Net model in PillowNet predicts one output at a time, we trained two separate models, one for denoising and one for peak calling. PillowNet is set up to run on CPUs, whereas AtacWorks runs only on GPUs. The high parallelism of GPUs allows for significantly faster training and prediction.

For this small training dataset, the total time required to train both PillowNet models was 85 minutes (39 minutes for the denoising model and 46 minutes for the peak classification model) with 64 CPU cores. On the other hand, we were able to use AtacWorks to train a single model performing both denoising and peak calling in 6 minutes using a single NVIDIA V100 GPU and in 4.5 minutes using 8 V100 GPUs on an NVIDIA DGX-1 server.

We were unable to train PillowNet on a larger dataset using the provided code, as the software did not succeed in loading the larger training dataset.

2. Comparison of the ResNet architecture used in AtacWorks with the U-Net architecture used in PillowNet

Since we were unable to apply the PillowNet code to chromosome-scale data, we instead re-implemented the U-Net architecture used in PillowNet in the AtacWorks framework. This allowed us to solely compare the default model architectures used by AtacWorks and PillowNet, in the same environment.

We were able to train U-Net models for denoising and peak calling on the aforementioned CD4+ T cell dataset with the U-Net architecture, using the loss functions and learning rate described[1]. We also trained a

standard AtacWorks ResNet model to perform both denoising and peak calling using default AtacWorks parameters (Supplementary Table 17).

The runtime for training using 8 Tesla V100 16GB GPUs was:

- AtacWorks ResNet: 185 seconds per epoch (regression + classification)
- U-Net (PillowNet reimplementation): 168 seconds per epoch (regression model) + 168 seconds per epoch (classification model)

We then applied the trained models to an ATAC-seq dataset from erythroblasts sampled to the same read depth.

We found that while the U-Net architecture performs well at both denoising and peak calling from this low-coverage dataset, the ResNet model performs better on all metrics (Supplementary Table 7).

We also note that PillowNet, as well as another previous method, Coda[2], train models that perform either denoising or peak calling, so that if both a denoised signal track and peak calls are needed, the user must train two independent models. The outputs of these two models need not be correlated with each other as the mappings they learn are independent of each other. On the other hand, AtacWorks performs denoising and peak calling jointly, so that the peak calls produced by AtacWorks from a noisy ATAC-seq dataset are a direct function of the denoised signal that it also produces.

3. Comparison of an AtacWorks ResNet model with an independently designed U-Net

To explore the suitability of U-Net architectures for denoising and peak calling from noisy ATAC-seq data, we also independently designed and tuned a U-Net architecture for these tasks. To be consistent with the framework of AtacWorks, our U-Net architecture differed from the U-Net architecture used in PillowNet in several ways:

a. We developed a single model for both denoising and peak calling.

b. Whereas PillowNet uses both the noisy ATAC-seq signal and peak calls from MACS2 as input for classification, our U-Net model uses only the noisy ATAC-seq signal.

c. Our model was trained with a joint MSE, Pearson correlation and BCE loss function, whereas PillowNet models are trained with MSE loss for regression and BCE loss for classification.

The runtime for training this independent U-Net model using 8 Tesla V100 16GB GPUs was:

- 254 seconds per epoch (regression + classification)

We tuned the hyperparameters of this model based on performance on the validation set consisting of a held-out chromosome. Performance was improved by using wider convolutional filters (25 bp compared to the 11 bp filters used in PillowNet). Nevertheless, the performance of this U-Net model on the task described above was comparable to that of the models based on the PillowNet architecture (Supplementary Table 7), and still did not match the performance of the ResNet model.

4. Note on ResNet and U-Net architectures

While we cannot rule out the possibility that a U-Net architecture designed and tuned in a specific way would outperform the ResNet architecture used in AtacWorks, we found that in all of our tests, the ResNet architecture consistently performed best on both denoising and peak calling tasks.

U-Net models have shown excellent performance on a variety of tasks in computer vision. We cannot give a definitive answer as to why we have observed the ResNet architecture to outperform U-Net on this particular application. We note a few possible reasons why the ResNet may be more suitable than the U-Net for the tasks here:

1. The U-Net model contains "max pooling" layers which reduce the size of the data representation by retaining only the maximum value across neighboring units, thus reducing resolution. The ResNet architecture does not include max pooling layers and does not compress the size of the data representation, instead using dilated convolutions to combine information over a large genomic distance.
2. The final layer of the U-Net combines very low-resolution features learned by the first layers of the model with high-resolution features spanning kilobases, which are learned by the final layers. This may not be ideal due to the very different scales of the features being combined. In the ResNet architecture, skip connections only skip every three convolutional layers, thus transferring information from shallower to deeper layers of the model without combining features of drastically different scales.

Supplementary Table 1

		**Pearson correlation**		**MSE**		**Spearman correlation**		**AUPRC**		**AUROC**		**Equivalent coverage**	
**Coverage (x million reads)**	**Region**	**Sub-sampled**	**Sub-sampled + AtacWorks**	**Sub-sampled**	**Sub-sampled + AtacWorks**	**Sub-sampled**	**Sub-sampled + AtacWorks**	**Sub-sampled + MACS2**	**Sub-sampled + AtacWorks**	**Sub-sampled +MACS2**	**Sub-sampled + AtacWorks**	**Based on Pearson correlation**	**Based on AUPRC**
0.2	Whole genome	0.2953	0.5324	124.4684	96.043	0.0987	0.1612	0.0347	0.1398	0.5322	0.6764	0.8	1.7
1	Whole genome	0.5735	0.8275	120.5494	41.6286	0.2056	0.3153	0.0977	0.311	0.5879	0.8167	3.8	6.1
5	Whole genome	0.8627	0.9478	101.9874	13.2582	0.4192	0.5441	0.2782	0.5048	0.7513	0.9173	11.5	14.8
10	Whole genome	0.9374	0.9659	81.0098	8.2864	0.5655	0.6531	0.4114	0.6055	0.8639	0.9524	15.2	21.6
20	Whole genome	0.9806	0.9813	46.3492	4.4977	0.747	0.7819	0.5856	0.7356	0.9545	0.9789	27	34.1
0.2	Chr10	0.2929	0.5446	120.5101	90.4098	0.0955	0.144	0.0328	0.1339	0.5175	0.6717		
1	Chr10	0.5483	0.8205	116.8774	40.4792	0.1993	0.2793	0.0885	0.2883	0.5708	0.8023		
5	Chr10	0.839	0.941	98.9833	13.0731	0.4107	0.5075	0.2673	0.4833	0.7406	0.9121		
10	Chr10	0.9143	0.9623	79.2786	8.3869	0.5548	0.6251	0.3962	0.5852	0.8556	0.9486		
20	Chr10	0.9661	0.9797	45.2997	4.5417	0.7381	0.7663	0.5674	0.7191	0.9503	0.9771		
0.2	All peaks	0.5001	0.5703	5258.5034	3449.5449	0.2556	0.3176						
1	All peaks	0.7941	0.854	5090.3765	1320.9353	0.4009	0.4814						
5	All peaks	0.9498	0.9632	4294.8691	349.4973	0.5557	0.615						
10	All peaks	0.9765	0.9813	3397.5979	180.4983	0.6451	0.6763						
20	All peaks	0.9911	0.9921	1921.0201	73.2704	0.7487	0.7567						
0.2	Non-peaks	0.0841	0.0966	14.1205	23.9647	0.0798	0.1372						
1	Non-peaks	0.19	0.2055	13.7308	14.1319	0.178	0.2896						
5	Non-peaks	0.4084	0.4829	11.868	6.0313	0.3906	0.5209						
10	Non-peaks	0.5618	0.6133	9.725	4.585	0.541	0.6331						
20	Non-peaks	0.743	0.763	6.0561	3.0196	0.7312	0.7682						
0.2	Differential peaks	0.3663	0.5072	1014.7882	776.6895	0.142	0.1874	0.657	0.7207	0.5083	0.5798		
1	Differential peaks	0.6682	0.8145	982.104	357.6288	0.2673	0.3376	0.6793	0.804	0.5409	0.6776		
5	Differential peaks	0.9004	0.9451	828.5622	116.9008	0.4855	0.5589	0.7722	0.8862	0.6723	0.8059		
10	Differential peaks	0.9508	0.9683	656.8795	66.8426	0.6277	0.674	0.8519	0.9238	0.7853	0.8701		
20	Differential peaks	0.9805	0.9852	373.5178	29.8795	0.7754	0.7892	0.9264	0.9595	0.8939	0.9313		

Supplementary Table 1: Performance of AtacWorks on bulk ATAC-seq data from human erythroblasts. ResNet models were trained on bulk ATAC-seq data from $CD4^+$ T cells, $CD8^+$ T cells, B cells, and NK cells. Metrics were calculated separately on the whole genome, on chromosome 10 (not used for training), and on differential

peaks (peaks present only in either the training data or the test data). MSE: Mean Squared Error. AUPRC: Area Under the Precision-Recall Curve. AUROC: Area Under the Receiver-Operator Characteristic.

Supplementary Table 2

		Pearson correlation			MSE			Spearman correlation		
Coverage (x million reads)	Region	Sub-sampled	Sub-sampled+ linear regression	Sub-sampled+ AtacWorks	Sub-sampled	Sub-sampled+ linear regression	Sub-sampled+ AtacWorks	Sub-sampled	Sub-sampled+ linear regression	Sub-sampled+ AtacWorks
0.2	Whole genome	0.2953	0.3501	0.5324	124.4684	120.7217	96.043	0.0987	0.1322	0.1612
1	Whole genome	0.5735	0.6341	0.8275	120.5494	83.6344	41.6286	0.2056	0.3167	0.3153
5	Whole genome	0.8627	0.8672	0.9478	101.9874	32.4167	13.2582	0.4192	0.4395	0.5441
10	Whole genome	0.9374	0.9318	0.9659	81.0098	17.0005	8.2864	0.5655	0.5759	0.6531
20	Whole genome	0.9806	0.9717	0.9813	46.3492	7.0099	4.4977	0.747	0.7479	0.7819
0.2	Chr10	0.2929	0.338	0.5446	120.5101	116.8217	90.4098	0.0955	0.1223	0.144
1	Chr10	0.5483	0.6064	0.8205	116.8774	85.5822	40.4792	0.1993	0.2798	0.2793
5	Chr10	0.839	0.8563	0.941	98.9833	33.1683	13.0731	0.4107	0.4253	0.5075
10	Chr10	0.9143	0.9208	0.9623	79.2786	17.7044	8.3869	0.5548	0.5622	0.6251
20	Chr10	0.9661	0.9673	0.9797	45.2997	7.1963	4.5417	0.7381	0.7385	0.7663
0.2	All peaks	0.5001	0.5478	0.5703	5258.5034	5099.5127	3449.5449	0.2556	0.3374	0.3176
1	All peaks	0.7941	0.8161	0.854	5090.3765	1489.5656	1320.9353	0.4009	0.4549	0.4814
5	All peaks	0.9498	0.9529	0.9632	4294.8691	386.7463	349.4973	0.5557	0.5762	0.615
10	All peaks	0.9765	0.9773	0.9813	3397.5979	187.2144	180.4983	0.6451	0.6529	0.6763
20	All peaks	0.9911	0.991	0.9921	1921.0201	74.2914	73.2704	0.7487	0.7487	0.7567
0.2	Non-peaks	0.0841	0.1099	0.0966	14.1205	13.7105	23.9647	0.0798	0.1028	0.1372
1	Non-peaks	0.19	0.2274	0.2055	13.7308	53.416	14.1319	0.178	0.2923	0.2896
5	Non-peaks	0.4084	0.4186	0.4829	11.868	24.801	6.0313	0.3906	0.4106	0.5209
10	Non-peaks	0.5618	0.5612	0.6133	9.725	13.342	4.585	0.541	0.5513	0.6331
20	Non-peaks	0.743	0.7361	0.763	6.0561	5.5637	3.0196	0.7312	0.732	0.7682

Supplementary Table 2: Comparison of AtacWorks and linear regression. Comparison of AtacWorks and linear regression models on bulk ATAC-seq data from human erythroblasts. The ResNet models in Supplementary Table 1 are compared against linear regression models for denoising, trained on the same data. MSE: Mean Squared Error.

Supplementary Table 3

		Pearson correlation			MSE			Spearman correlation			AUPRC			AUROC		
Coverage (x million reads)	Region	Sub-sampled	Sub-sampled +AW (1 cell type)	Sub-Sampled +AW (4 cell types)	Sub-sampled	Sub-Sampled +AW (1 cell type)	Sub-Sampled +AW (4 cell types)	Sub-sampled	Sub-Sampled +AW (1 cell type)	Sub-Sampled +AW (4 cell types)	Sub-Sampled + MACS2	Sub-Sampled +AW (1 cell type)	Sub-Sampled +AW (4 cell types)	Sub-sampled +MACS2	Sub-sampled +AW (1 cell type)	Sub-sampled +AW (4 cell types)
0.2	Whole genome	0.2953	0.5241	0.5324	124.4684	108.6078	96.043	0.0987	0.1534	0.1612	0.0347	0.1351	0.1398	0.5322	0.6704	0.6764
1	Whole genome	0.5735	0.8265	0.8275	120.5494	42.6534	41.6286	0.2056	0.2976	0.3153	0.0977	0.3076	0.311	0.5879	0.8107	0.8167
5	Whole genome	0.8627	0.9458	0.9478	101.9874	14.2922	13.2582	0.4192	0.5366	0.5441	0.2782	0.4951	0.5048	0.7513	0.9124	0.9173
10	Whole genome	0.9374	0.9654	0.9659	81.0098	8.3982	8.2864	0.5655	0.6482	0.6531	0.4114	0.6038	0.6055	0.8639	0.9508	0.9524
20	Whole genome	0.9806	0.9813	0.9813	46.3492	4.5704	4.4977	0.747	0.779	0.7819	0.5856	0.7335	0.7356	0.9545	0.9789	0.9789
0.2	Chr10	0.2929	0.5317	0.5446	120.5101	102.9197	90.4098	0.0955	0.1384	0.144	0.0328	0.1299	0.1339	0.5175	0.6667	0.6717
1	Chr10	0.5483	0.8177	0.8205	116.8774	41.535	40.4792	0.1993	0.2663	0.2793	0.0885	0.2852	0.2883	0.5708	0.7965	0.8023
5	Chr10	0.839	0.9386	0.941	98.9833	13.9441	13.0731	0.4107	0.501	0.5075	0.2673	0.475	0.4833	0.7406	0.9075	0.9121
10	Chr10	0.9143	0.9609	0.9623	79.2786	8.5287	8.3869	0.5548	0.6196	0.6251	0.3962	0.5838	0.5852	0.8556	0.9468	0.9486
20	Chr10	0.9661	0.9791	0.9797	45.2997	4.6037	4.5417	0.7381	0.7632	0.7663	0.5674	0.7172	0.7191	0.9503	0.977	0.9771
0.2	All peaks	0.5001	0.5694	0.5703	5258.5034	3677.3535	3449.5449	0.2556	0.3227	0.3176						
1	All peaks	0.7941	0.8519	0.854	5090.3765	1352.3778	1320.9353	0.4009	0.4782	0.4814						
5	All peaks	0.9498	0.9621	0.9632	4294.8691	366.5408	349.4973	0.5557	0.6081	0.615						
10	All peaks	0.9765	0.9807	0.9813	3397.5979	181.6377	180.4983	0.6451	0.6724	0.6763						
20	All peaks	0.9911	0.9919	0.9921	1921.0201	75.5221	73.2704	0.7487	0.7543	0.7567						
0.2	Non-peaks	0.0841	0.0865	0.0966	14.1205	31.9033	23.9647	0.0798	0.1284	0.1372						
1	Non-peaks	0.19	0.2028	0.2055	13.7308	14.503	14.1319	0.178	0.271	0.2896						
5	Non-peaks	0.4084	0.4699	0.4829	11.868	6.7211	6.0313	0.3906	0.5132	0.5209						
10	Non-peaks	0.5618	0.6112	0.6133	9.725	4.6747	4.585	0.541	0.6281	0.6331						
20	Non-peaks	0.743	0.7542	0.763	6.0561	3.0454	3.0196	0.7312	0.765	0.7682						

Supplementary Table 3: Performance of AtacWorks on bulk ATAC-seq data from human erythroblasts using different training datasets. ResNet models trained on bulk ATAC-seq data from 4 cell types (Supplementary Table 1) are compared against ResNet models trained on bulk ATAC-seq data from 1 cell type ($CD4^+$ T cells). MSE: Mean Squared Error. AUPRC: Area Under the Precision-Recall Curve. AUROC: Area Under the Receiver-Operator Characteristic. AW: AtacWorks.

Supplementary Table 4

		Pearson correlation			MSE			AUPRC			AUROC		
Coverage (x million reads)	Region	Sub-sampled	Sub-sampled+ AW (4 blood cell types)	Sub-sampled +AW (ENCODE 3 tissues)	Sub-sampled	Sub-Sampled +AW (4 blood cell types)	Sub-sampled+ AW (ENCODE 3 tissues)	Sub-sampled +MACS2	Sub-Sampled +AW (4 blood cell types)	Sub-Sampled +AW (ENCODE 3 tissues)	Sub-sampled+ MACS2	Sub-Sampled +AW (4 blood cell types)	Sub-sampled +AW (ENCODE 3 tissues)
0.2	Whole genome	0.1886	0.5159	0.5996	42.1937	38.8961	29.5009	0.0179	0.0486	0.049	0.5216	0.6317	0.6322
1	Whole genome	0.3573	0.7012	0.7501	40.9502	23.2419	19.2466	0.0396	0.1659	0.1803	0.5634	0.7875	0.7908
5	Whole genome	0.6623	0.8697	0.8683	34.8418	8.9874	8.4242	0.177	0.4269	0.4412	0.7318	0.9123	0.9153
10	Whole genome	0.7943	0.9075	0.909	27.9176	6.1835	5.9574	0.3077	0.5526	0.5692	0.8521	0.952	0.9539
20	Whole genome	0.9061	0.9445	0.944	16.3667	3.7409	3.6545	0.5058	0.707	0.7137	0.9488	0.9808	0.9811
0.2	Chr10	0.1136	0.1689	0.1743	28.063	40.482	25.7791	0.0188	0.0439	0.0446	0.5095	0.602	0.6024
1	Chr10	0.2424	0.3891	0.4112	27.2533	25.2848	19.5273	0.0366	0.1409	0.1536	0.5491	0.7571	0.7591
5	Chr10	0.505	0.6858	0.6942	23.3745	9.6798	8.7078	0.1658	0.3932	0.4071	0.7192	0.8935	0.8968
10	Chr10	0.6605	0.778	0.7843	18.9262	6.7641	6.2687	0.2955	0.5164	0.5318	0.8405	0.9403	0.9424
20	Chr10	0.8215	0.8679	0.8697	11.4164	4.1109	3.9582	0.4825	0.6726	0.6798	0.9411	0.9756	0.9763

Supplementary Table 4: Performance of AtacWorks on ENCODE bulk ATAC-seq data from the human Peyer's Patch. The ResNet models in Supplementary Table 1 are compared against ResNet models trained on data from three human tissue samples (coronary artery, tibial nerve, and left ventricle) sequenced as part of the ENCODE project. MSE: Mean Squared Error. AUPRC: Area Under the Precision-Recall Curve. AUROC: Area Under the Receiver-Operator Characteristic. AW: AtacWorks.

Supplementary Table 5

Donor/replicate	# of reads	Visual TSS score
6792-7A	8,885,084	10
6792-7B	9,399,272	12
**Mono-7.low_qual.bam**	**18,284,356**	**11**
4983-7A	8,426,328	14
4983-7B	6,672,448	20
Donor7256-7A-141106	16,498,592	15
Donor7256-7B-141106	19,930,272	14
**Mono-7.high_qual.bam**	**51,527,640**	**16**
Donor2596-Erythroblast-15A-150303	14,444,098	5
Donor2596-Erythroblast-15B-150303	6,414,806	8
**Erythro-15.low_qual.bam**	**20,858,904**	**6**
Donor2596-Erythroblast-15C-150303	17,261,154	10
Donor5483-Erythroblast-15A-150305	3,271,174	20
Donor5483-Erythroblast-15B-150305	1,775,816	19
Donor5483-Erythroblast-15C-150305	3,534,964	10
Donor6926-Erythroblast-15A-150318	3,534,964	20
Donor6926-Erythroblast-15B-150318	4,806,218	19
**Erythro-15.high_qual.bam**	**34,184,290**	**13**

Supplementary Table 5: Aggregation of bulk ATAC-seq tracks by quality. Breakdown of how human monocyte and erythroblast ATAC-seq tracks were aggregated across donors and replicates to create paired high and low quality training data.

Supplementary Table 6

	Pearson correlation		MSE		Spearman correlation		AUPRC		AUROC	
**Region**	**Low quality**	**Low quality + AtacWorks**	**Low quality**	**Low quality + AtacWorks**	**Low quality**	**Low quality + AtacWorks**	**Low quality + MACS2**	**Low quality + AtacWorks**	**Low quality + MACS2**	**Low quality + AtacWorks**
Whole genome	0.7056	0.8952	18.874	11.3718	0.2654	0.3307	0.4121	0.2684	0.8747	0.8096
Chr10	0.6891	0.9066	18.2168	10.5776	0.2361	0.282	0.2407	0.3816	0.7892	0.8573
All peaks	0.9114	0.9212	967.2128	601.3749	0.6048	0.6492				
Non-peaks	0.224	0.2202	3.7774	1.9796	0.2414	0.3028				

Supplementary Table 6: Performance of AtacWorks on low-quality bulk ATAC-seq signal from human erythroblasts. A ResNet model was trained on paired low- and high-quality ATAC-seq data from human monocytes. MSE: Mean Squared Error. AUPRC: Area Under the Precision-Recall Curve. AUROC: Area Under the Receiver-Operator Characteristic.

Supplementary Table 7

Region	Coverage (x million reads)	1	1	1	1
		**Whole genome**	**Chr10**	**All peaks**	**Non-peaks**
**Pearson Correlation**	Subsampled	0.5735	0.5483	0.7941	0.19
	Subsampled+ AtacWorks resnet	0.8265	0.8177	0.8519	0.2028
	Subsampled+ PillowNet U-net	0.7825	0.7728	0.8338	0.1936
	Subsampled + U-net 2	0.7784	0.7716	0.8375	0.1046
**MSE**	Subsampled	120.5494	116.8774	5090.3765	13.7308
	Subsampled+ AtacWorks resnet	42.6534	41.535	1352.3778	14.503
	Subsampled+ PillowNet U-net	44.8554	44.36	1446.2043	14.7357
	Subsampled + U-net 2	119.8178	116.2994	5038.1045	14.107
**Spearman Correlation**	Subsampled	0.2056	0.1993	0.4009	0.178
	Subsampled+ AtacWorks resnet	0.2976	0.2663	0.4782	0.271
	Subsampled+ PillowNet U-net	0.233	0.218	0.4634	0.2003
	Subsampled + U-net 2	0.1381	0.1336	0.465	0.0934
**AUPRC**	Subsampled	0.0977	0.0885		
	Subsampled+ AtacWorks resnet	0.3076	0.2852		
	Subsampled+ PillowNet U-net	0.1272	0.1181		
	Subsampled + U-net 2	0.1919	0.1726		
**AUROC**	Subsampled	0.5879	0.5708		
	Subsampled+ AtacWorks resnet	0.8107	0.7965		
	Subsampled+ PillowNet U-net	0.7243	0.7428		
	Subsampled + U-net 2	0.7783	0.7623		

Supplementary Table 7: Comparison of AtacWorks and U-Net on bulk ATAC-seq data from erythroblasts. ResNet models are compared against a previously-described U-Net model architecture[1], as well as an independently developed U-Net architecture. All models were trained on bulk ATAC-seq data from human $CD4^+$ T cells. MSE: Mean Squared Error. AUPRC: Area Under the Precision-Recall Curve. AUROC: Area Under the Receiver-Operator Characteristic.

Supplementary Table 8

			Pearson correlation		MSE		Spearman correlation		AUPRC		AUROC		Equivalent #cells	
**Cells**	**Approx coverage (x million reads)**	**Region**	**Sub-sampled**	**Sub-sampled +AW**	**Sub-sampled**	**Sub-sampled +AW**	**Sub-sampled**	**Sub-sampled +AW**	**Sub-sampled +MACS2**	**Sub-sampled +AW**	**Sub-sampled +MACS2**	**Sub-sampled +AW**	**Based on Pearson correlation**	**Based on AUPRC**
5	0.1	Whole genome	0.6922	0.7617	1291.1573	547.6562	0.1411	0.2596	0.0361	0.3983	0.5438	0.7563	9	128
10	0.2	Whole genome	0.7783	0.8002	1285.0303	465.422	0.1872	0.2333	0.0649	0.4895	0.5718	0.8139	12	183
50	1	Whole genome	0.9328	0.9496	1246.7446	128.0476	0.287	0.3631	0.2048	0.7008	0.6561	0.9292	67	406
5	0.1	Chr10	0.6589	0.7427	981.3273	448.0301	0.1347	0.238	0.0363	0.3694	0.5238	0.7423		
10	0.2	Chr10	0.7513	0.7779	976.6679	389.8831	0.1779	0.2218	0.0601	0.4587	0.5615	0.799		
50	1	Chr10	0.9244	0.9459	947.8414	104.3384	0.2793	0.3387	0.2107	0.6748	0.6402	0.9191		
5	0.1	All peaks	0.686	0.7346	68151.6953	28093.5059	0.4292	0.4685						
10	0.2	All peaks	0.7665	0.7776	67828.3047	24047.6816	0.5088	0.5304						
50	1	All peaks	0.9288	0.9418	65807.0625	6643.3594	0.6751	0.7119						
5	0.1	Non-peaks	0.0687	0.1558	1.1033	16.1673	0.0634	0.1723						
10	0.2	Non-peaks	0.1103	0.1187	1.0976	10.4094	0.0907	0.1181						
50	1	Non-peaks	0.2059	0.3245	1.0726	2.3366	0.1751	0.2753						

Supplementary Table 8: Performance of AtacWorks on dscATAC data from human NK cells. ResNet models were trained on dscATAC data from human B cells and monocytes. MSE: Mean Squared Error. AUPRC: Area Under the Precision-Recall Curve. AUROC: Area Under the Receiver-Operator Characteristic. dscATAC: Droplet Single-Cell ATAC-seq. NK: Natural Killer. AW: AtacWorks.

Supplementary Table 9

			Pearson correlation		MSE		Spearman correlation		AUPRC		AUROC		Equivalent # cells	
**Barcode**	**Coverage (reads)**	**Region**	**Sub-sampled**	**Sub-sampled +AW**	**Sub-sampled**	**Sub-sampled +AW**	**Sub-sampled**	**Sub-sampled +AW**	**Sub-sampled +MACS2**	**Sub-sampled +AW**	**Sub-sampled +MACS2**	**Sub-sampled +AW**	**Based on Pearson correlation**	**Based on AUPRC**
aattggaggt tagaaata	20012	Whole genome	0.4008	0.479	1296.5016	998.5561	0.0754	0.1712	0.0202	0.1804	0.5228	0.612	1-2	21
ctaggtcacc actgcggt	19990	Whole genome	0.3951	0.4679	1296.4674	1011.2458	0.0738	0.1704	0.0206	0.1769	0.5183	0.6137	1-2	21
gtacaggat caaatccgg	19988	Whole genome	0.3904	0.4687	1296.5342	1009.9517	0.0761	0.1752	0.0202	0.1874	0.5206	0.6178	1-2	23
gtggtgggg atctgtgta	19984	Whole genome	0.3522	0.4196	1296.5994	1073.1012	0.0724	0.1743	0.0212	0.1685	0.5194	0.6127	1-2	20
aattggaggt tagaaata	20012	Chr10	0.3591	0.432	985.4442	802.4686	0.0708	0.148	0.0187	0.1458	0.5031	0.5946		
ctaggtcacc actgcggt	19990	Chr10	0.3609	0.4324	985.3823	804.9632	0.0662	0.1466	0.0221	0.1386	0.525	0.5923		
gtacaggat caaatccgg	19988	Chr10	0.3941	0.47	985.2795	767.851	0.0746	0.1684	0.0229	0.1947	0.5108	0.6222		
gtggtgggg atctgtgta	19984	Chr10	0.354	0.4161	985.4137	826.1671	0.0661	0.1576	0.0211	0.147	0.4948	0.5977		
aattggaggt tagaaata	20012	All peaks	0.3922	0.4447	68433.8594	51679.5508	0.2429	0.2551						
ctaggtcacc actgcggt	19990	All peaks	0.3911	0.4369	68432.0547	51895.7461	0.2369	0.2474						
gtacaggat caaatccgg	19988	All peaks	0.3777	0.4293	68435.5781	52237.9648	0.2408	0.2562						
gtggtgggg atctgtgta	19984	All peaks	0.3488	0.3905	68439.0391	54451.7227	0.2223	0.2337						
aattggaggt tagaaata	20012	Non-peaks	0.0331	0.094	1.1065	20.6814	0.0292	0.1094						
ctaggtcacc actgcggt	19990	Non-peaks	0.0333	0.0855	1.1064	29.4447	0.0308	0.1091						
gtacaggat caaatccgg	19988	Non-peaks	0.0326	0.0957	1.1065	21.5226	0.0296	0.1119						
gtggtgggg atctgtgta	19984	Non-peaks	0.0373	0.0862	1.1061	43.1767	0.0326	0.1149						

Supplementary Table 9: Performance of AtacWorks on denoising and peak calling from single cells. A ResNet model was trained on droplet single-cell ATAC-seq (dscATAC) data from human B cells and monocytes, and was applied to 4 different human natural killer (NK) cells. MSE: Mean Squared Error. AUPRC: Area Under the Precision-Recall Curve. AUROC: Area Under the Receiver-Operator Characteristic. AW: AtacWorks.

Supplementary Table 10

			Pearson correlation		MSE		Spearman correlation		AUPRC		AUROC	
**Cells**	**Approx coverage (x million reads)**	**Region**	**Sub-sampled**	**Sub-sampled+ AtacWorks**	**Sub-sampled**	**Sub-sampled+ AtacWorks**	**Sub-sampled**	**Sub-sampled+ AtacWorks**	**Sub-sampled +MACS2**	**Sub-sampled+ AtacWorks**	**Sub-sampled +MACS2**	**Sub-sampled+ AtacWorks**
450	1	Whole genome	0.972	0.9737	2801.6499	152.76	0.3347	0.3896	0.3161	0.7552	0.7033	0.9451
450	1	Chr10	0.967	0.969	2150.2637	137.6873	0.3222	0.3608	0.3026	0.7409	0.6862	0.9418
450	1	All peaks	0.9694	0.9699	198294.5469	10640.4434	0.744	0.7394				
450	1	Non-peaks	0.2274	0.3857	0.543	2.4879	0.1844	0.2912				

Supplementary Table 10: Performance of AtacWorks on dsciATAC data from human CD4$^+$ T cells. The ResNet model used was trained on groups of 50 human blood cells sequenced using the droplet single-cell ATAC-seq (dscATAC) protocol. dsciATAC: Droplet-based Single-cell Combinatorial Indexing ATAC-seq.

Supplementary Table 11

	**Number of Cells**	**35**	**35**	**35**	**35**
	**Approx coverage (x million reads)**	**1**	**1**	**1**	**1**
**Region**		**Whole genome**	**Chr10**	**All peaks**	**Non-peaks**
**Pearson correlation**	Subsampled	0.8801	0.8511	0.8869	0.3026
	Subsampled + AtacWorks (dscATAC-seq)	0.917	0.8947	0.9015	0.3197
	Subsampled +AtacWorks (sciATAC-seq)	0.9271	0.8987	0.9134	0.3697
**MSE**	Subsampled	96.7348	74.4388	4267.9238	0.9885
	Subsampled +AtacWorks (dscATAC-seq)	70.9418	54.1613	3120.0884	0.9511
	Subsampled +AtacWorks (sciATAC-seq)	15.8201	16.0614	639.1514	1.512
**Spearman correlation**	Subsampled	0.3609	0.3655	0.6488	0.2724
	Subsampled +AtacWorks (dscATAC-seq)	0.3815	0.3884	0.6668	0.2708
	Subsampled +AtacWorks (sciATAC-seq)	0.3761	0.3806	0.6638	0.2832
**AUPRC**	Subsampled + MACS2	0.3391	0.3404		
	Subsampled +AtacWorks (dscATAC-seq)	0.7332	0.7332		
	Subsampled +AtacWorks (sciATAC-seq)	0.7483	0.7556		
**AUROC**	Subsampled + MACS2	0.7159	0.7167		
	Subsampled +AtacWorks (dscATAC-seq)	0.9555	0.9555		
	Subsampled +AtacWorks (sciATAC-seq)	0.9604	0.9614		

Supplementary Table 11: Performance of AtacWorks on sciATAC data from macrophages in a mouse lung tumor sample. A ResNet model trained on groups of 50 human blood cells sequenced using the droplet single-cell ATAC-seq (dscATAC) protocol is compared against a model trained on mouse B cells and Monocytes in the same sciATAC dataset. MSE: Mean Squared Error. AUPRC: Area Under the Precision-Recall Curve. AUROC: Area Under the Receiver-Operator Characteristic. sciATAC: Single-cell Combinatorial Indexing ATAC-seq.

Supplementary Table 12

		Pearson correlation		MSE		Spearman correlation	
Coverage (x million reads)	Region	Sub-sampled	Sub-sampled+ AtacWorks	Sub-sampled	Sub-sampled+ AtacWorks	Sub-sampled	Sub-sampled+ AtacWorks
1	CTCF Motif regions - Whole genome	0.5346	0.7199	86.0941	41.0938	0.2573	0.4703
5	CTCF Motif regions - Whole genome	0.8198	0.8764	79.3968	19.5325	0.4742	0.6046
10	CTCF Motif regions - Whole genome	0.9018	0.9192	71.3574	13.0298	0.5851	0.6696
20	CTCF Motif regions - Whole genome	0.9524	0.9604	56.628	8.4881	0.7004	0.7269
1	CTCF Motif regions - Chr10	0.5186	0.7187	75.9466	36.1097	0.2561	0.4865
5	CTCF Motif regions - Chr10	0.8108	0.8839	69.9755	16.1752	0.4719	0.6213
10	CTCF Motif regions - Chr10	0.8941	0.9306	62.9949	9.8819	0.5838	0.6889
20	CTCF Motif regions - Chr10	0.9492	0.9616	49.8286	5.5942	0.6986	0.7486

Supplementary Table 12: Performance of AtacWorks at CTCF binding sites. The performance of AtacWorks was measured at CTCF binding sites in high-resolution bulk ATAC-seq data from hematopoietic stem cells (HSCs). ResNet models were trained using high-resolution bulk ATAC-seq data from multipotent progenitor (MPP) cells, CD8+ T cells, and NK cells. Both the training and test set were limited to 200-bp regions surrounding CTCF motifs.

Supplementary Table 13

full_name	name	variability	bootstrap_lower_bound	bootstrap_upper_bound	p_value	p_value_adj
**Long-term HSCs**						
ENSG00000185697_LINE2870_MYBL1_D_N1	MYBL1	1.067342746	0.9791370197	1.157565799	2.40E-21	2.67E-18
ENSG00000101216_LINE3292_GMEB2_D_N1	GMEB2	1.067164574	0.6189915519	1.351010569	3.04E-21	2.67E-18
ENSG00000101057_LINE2838_MYBL2_D_N1	MYBL2	1.058762737	1.00184957	1.114064289	9.72E-17	5.70E-14
ENSG00000197576_LINE16121_HOXA4_I_N1	HOXA4	1.039082223	0.9826564613	1.095651818	2.02E-08	8.88E-06
ENSG00000101412_LINE1750_E2F1_D_N1	E2F1	1.03851618	0.8014084025	1.229382582	3.15E-08	1.11E-05
ENSG00000124664_LINE1834_SPDEF_D_N2	SPDEF	1.028839055	0.8297630601	1.219492347	2.47E-05	0.007245619449
**Lymphoid-primed HSCs**						
ENSG00000101412_LINE1749_E2F1_D_N1	E2F1	1.342320427	0	1.893766984	0	0
ENSG00000214717_LINE3702_ZBED1_D	ZBED1	1.145643841	0.9578956753	1.29824065	2.54E-90	2.24E-87
ENSG00000169953_LINE2711_HSFY2_D_N1	HSFY2	1.100473495	0.8613554596	1.306172853	1.16E-44	6.81E-42
ENSG00000169953_LINE2710_HSFY2_D_N2	HSFY2	1.078623847	0.7330218719	1.318083244	2.65E-28	9.34E-26
ENSG00000172468_LINE2715_HSFY1_I	HSFY1	1.078623847	0.7330218719	1.318083244	2.65E-28	9.34E-26
ENSG00000101057_LINE2836_MYBL2_D_N1	MYBL2	1.047042805	0.9146271082	1.162710284	2.08E-11	6.12E-09
ENSG00000139515_LINE2371_PDX1_D_N2	PDX1	1.031957017	1.005817911	1.055337692	3.49E-06	0.0008778262953
ENSG00000185697_LINE2871_MYBL1_D_N1	MYBL1	1.028741457	0.9215863123	1.12120042	2.62E-05	0.005767417033
**Erythroid-primed HSCs**						
ENSG00000171532_LINE264_NEUROD2_D	NEUROD2	1.308414561	0.3020714671	1.833942362	0	0
ENSG00000170370_LINE2476_EMX2_D_N1	EMX2	1.079749775	0.9790324421	1.16608414	4.71E-29	4.15E-26
ENSG00000113430_LINE2265_IRX4_I	IRX4	1.035674077	0.8818873262	1.181428961	2.67E-07	0.0001174700987
ENSG00000159387_LINE2406_IRX6_I	IRX6	1.035674077	0.8818873262	1.181428961	2.67E-07	0.0001174700987
ENSG00000105991_LINE2206_HOXA1_D_N1	HOXA1	1.031548681	0.8952648341	1.147233213	4.55E-06	0.001602671285

Supplementary Table 13: Differentially-accessible motifs in putative regulatory regions. Differentially-accessible transcription factor motifs within sets of putative lineage-priming regulatory elements identified using chromVAR[3].

Supplementary Table 14

Parameter	Default	CTCF footprinting
**Length of input genomic intervals**	50,000 bp	200 bp
**Padding on either side of genomic intervals**	5,000 bp	200 bp
**Number of residual blocks**	7 total (5 followed by regression output, then 2 more followed by classification output)	
**Kernel size**	51 (for all convolutional layers)	11 (for all convolutional layers)
**Dilation**	8 (for all convolutional layers)	1 (for all convolutional layers)
**Number of channels**	15 (for all convolutional layers)	32 (for all convolutional layers)
**Batch size**	64	
**Weights for loss functions:**		
Mean squared error	0.0005	0.008
1 - Pearson correlation coefficient	1	20
Binary cross-entropy	1	1
**Metric to choose best model**	AUROC	Pearson correlation

Supplementary Table 14: Model parameters. Parameters used to train the AtacWorks ResNet models described in this paper.

Supplementary Figure 1

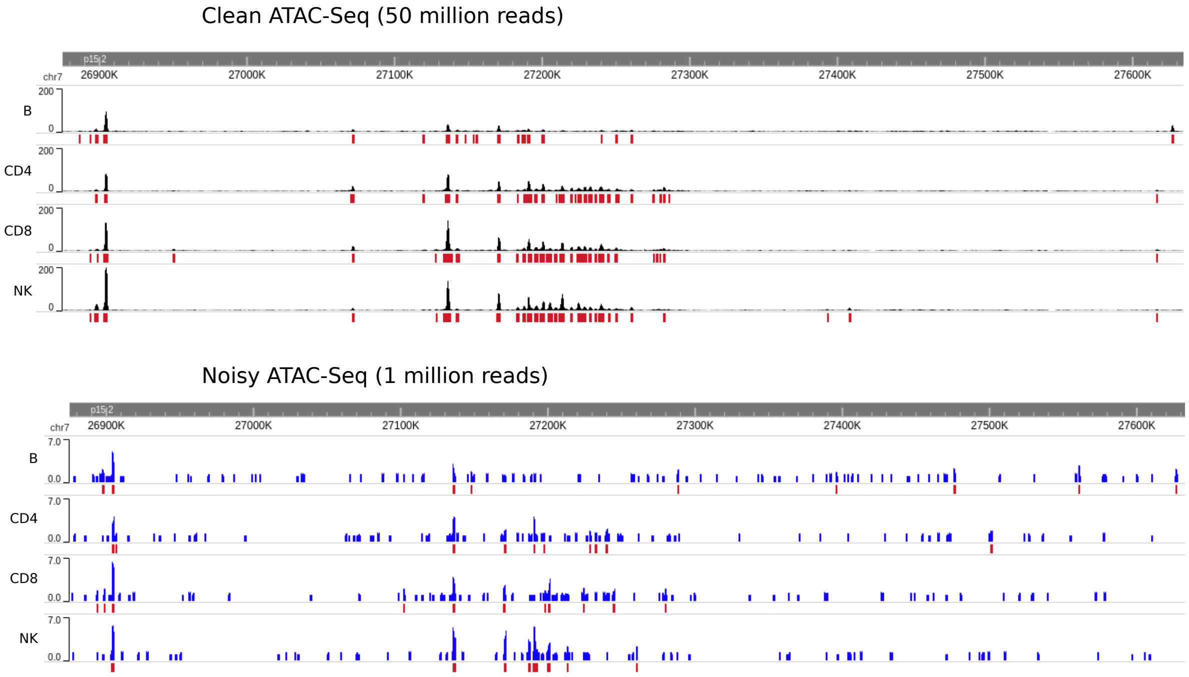

Supplementary Figure 1: Bulk ATAC-seq data used to train AtacWorks. Clean (black) and noisy (blue) ATAC-seq signal tracks for the 4 ATAC-seq datasets (CD4+ T cells, CD8+ T cells, B cells and NK cells) used for training the deep learning model. NK cells: Natural Killer cells.

Supplementary Figure 2

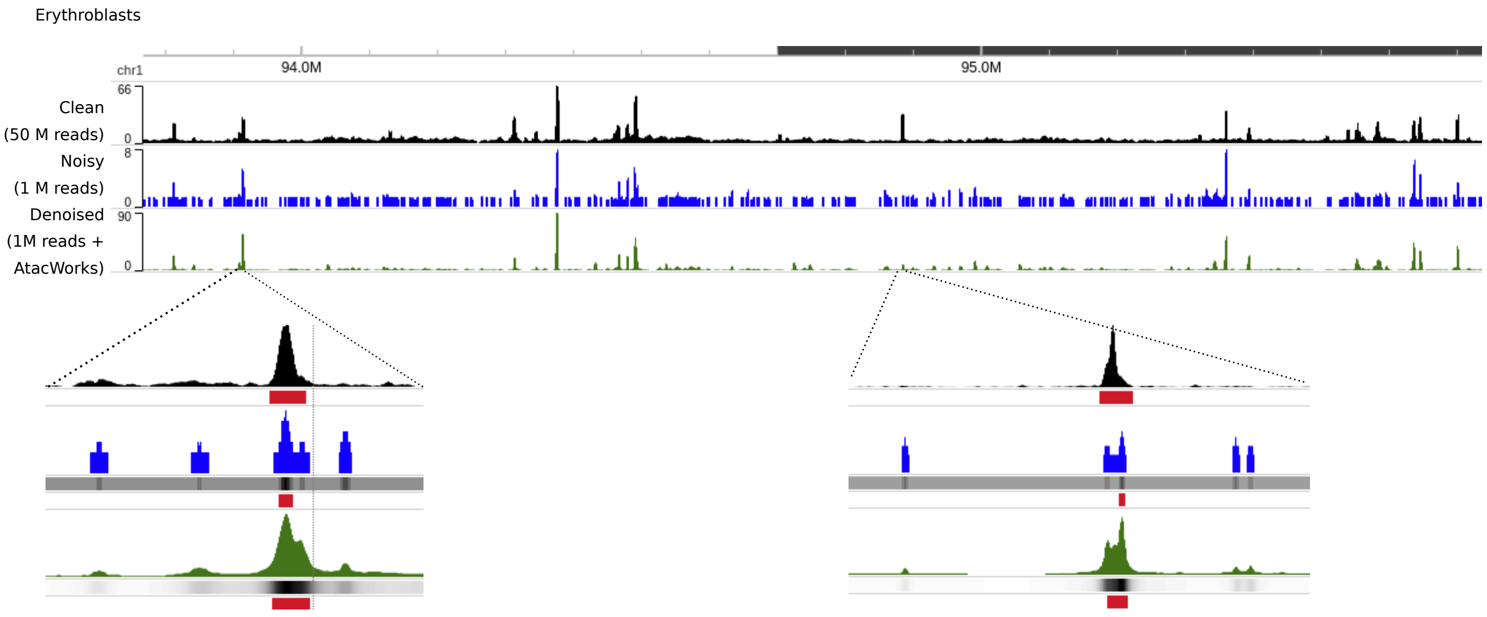

Supplementary Figure 2: AtacWorks denoises low-coverage bulk ATAC-seq from human erythroblasts. Clean (black), noisy (blue) and denoised (green) ATAC-seq signal tracks for bulk ATAC-seq data from erythroid cells. Detailed views of two peaks are shown. Below the noisy (blue) signal tracks, the heatmaps show the negative log of the p-value returned by MACS2 for each position and the red bars show peak calls by MACS2 using a p-value cutoff of 10^{-3}. Below the denoised (green) signal tracks, the heatmaps show the probability returned by AtacWorks (representing the probability that each position is part of a peak) and the red bars show peak calls by AtacWorks using a probability cutoff of 0.5.

Supplementary Figure 3

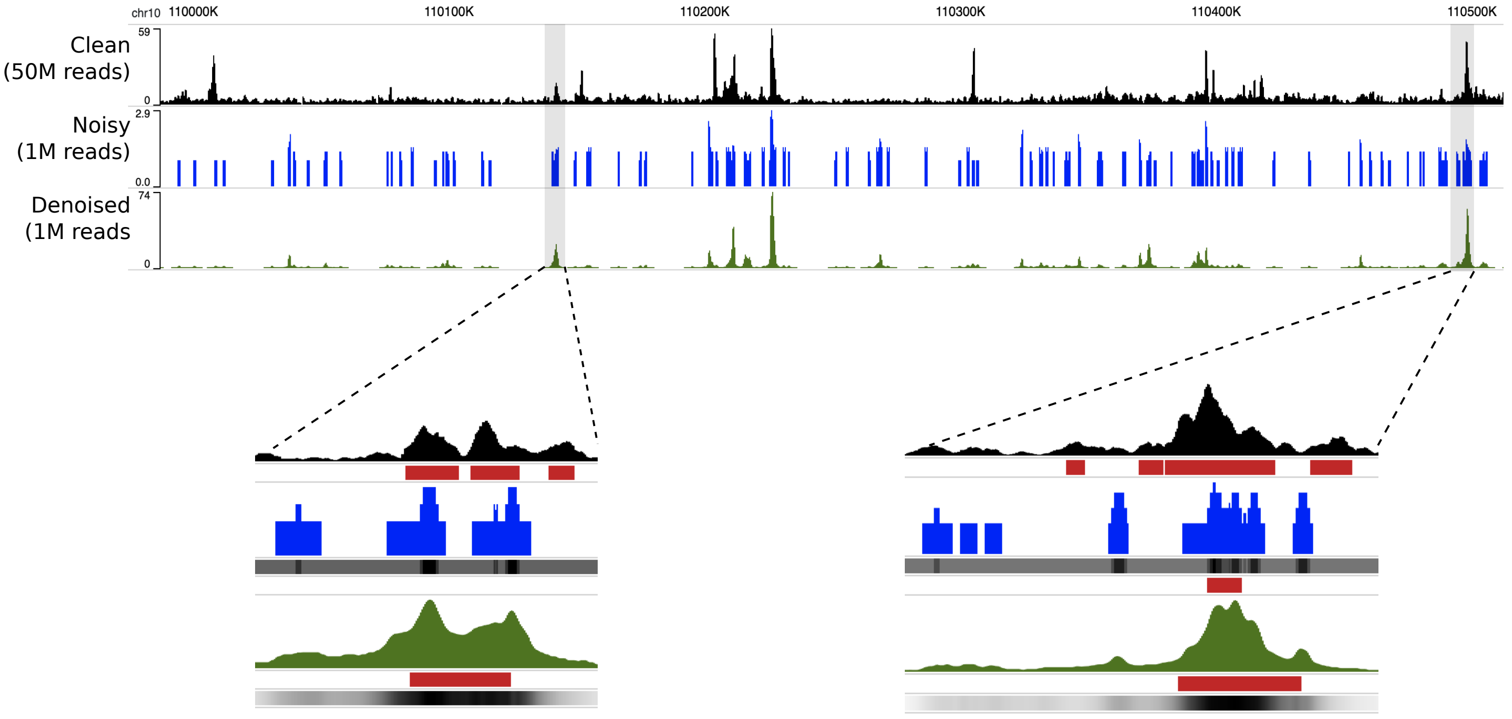

Supplementary Figure 3: AtacWorks denoises low-coverage bulk ATAC-seq data from the human Peyer's Patch. Clean (black), noisy (blue) and denoised (green) ATAC-seq signal tracks for ENCODE bulk ATAC-seq data from the Peyer's Patch. Detailed views of two regions are shown. Below the noisy (blue) signal tracks, the heatmaps show the negative log of the p-value returned by MACS2 for each position and the red bars show peak calls by MACS2 using a p-value cutoff of 10^{-3}. Below the denoised (green) signal tracks, the heatmaps show the probability returned by AtacWorks (representing the probability that each position is part of a peak) and the red bars show peak calls by AtacWorks using a probability cutoff of 0.5.

Supplementary Figure 4

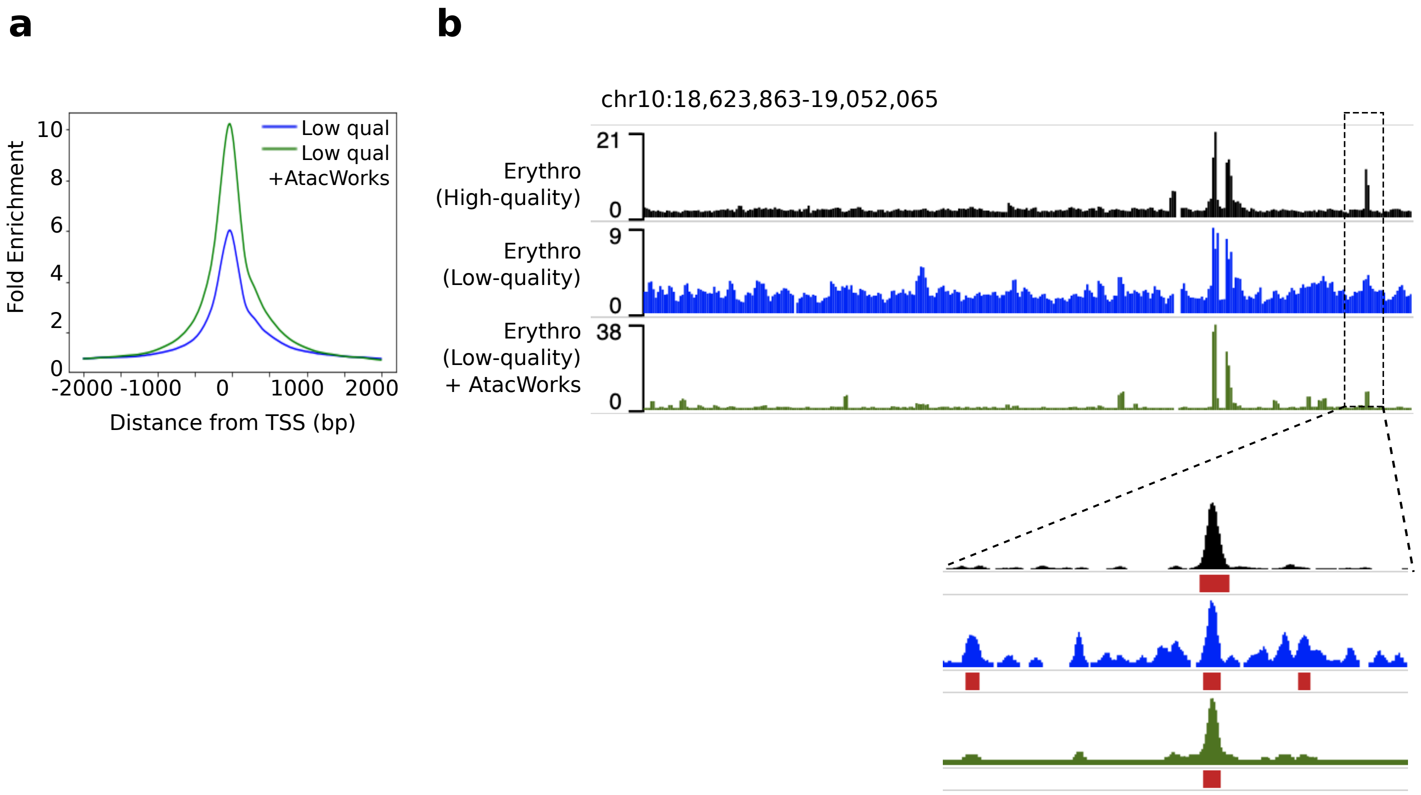

Supplementary Figure 4: AtacWorks improves the signal-to-noise ratio in low-quality ATAC-seq. (a) Average ATAC-seq signal over 4000-bp windows centered on transcription start sites, in low-quality ATAC-seq data from erythroblasts, before (blue) and after (green) denoising with AtacWorks. (b) ATAC-seq signal tracks for high-quality (black) and low-quality (blue) data from erythroblasts, and low-quality data after denoising by AtacWorks (green). Red bars below the tracks show peak calls by MACS2 (for the high and low-quality tracks) and AtacWorks (for the denoised track). Source data are provided as a Source Data file.

Supplementary Figure 5

a

b

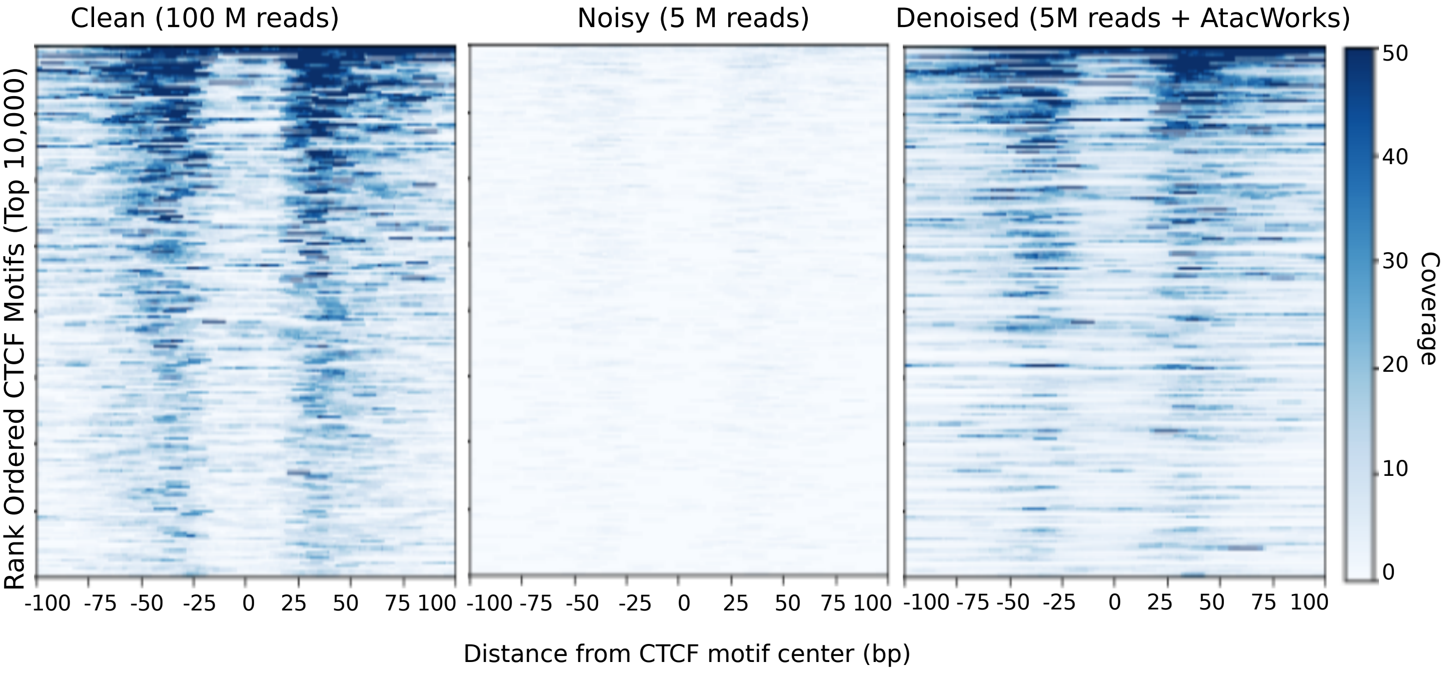

Supplementary Figure 5: Enhancing transcription factor footprints with AtacWorks. (a) Signal tracks around a CTCF binding site in HSCs. From top to bottom: CTCF ChIP-seq signal (yellow), ATAC-seq signal at a depth of 100 million reads (black), subsampled to 5 million reads (blue), and subsampled signal denoised by AtacWorks (green). The black bar underneath the ChIP-seq track shows the CTCF binding motif. (b) Heatmaps showing the signal at 10,000 genomic regions surrounding CTCF motifs (rows) in clean (100 million read), noisy (downsampled to 5 million reads) and denoised signals. Color intensity represents sequencing coverage. CTCF: CCCTC-binding factor. Source data are provided as a Source Data file.

Supplementary Figure 6

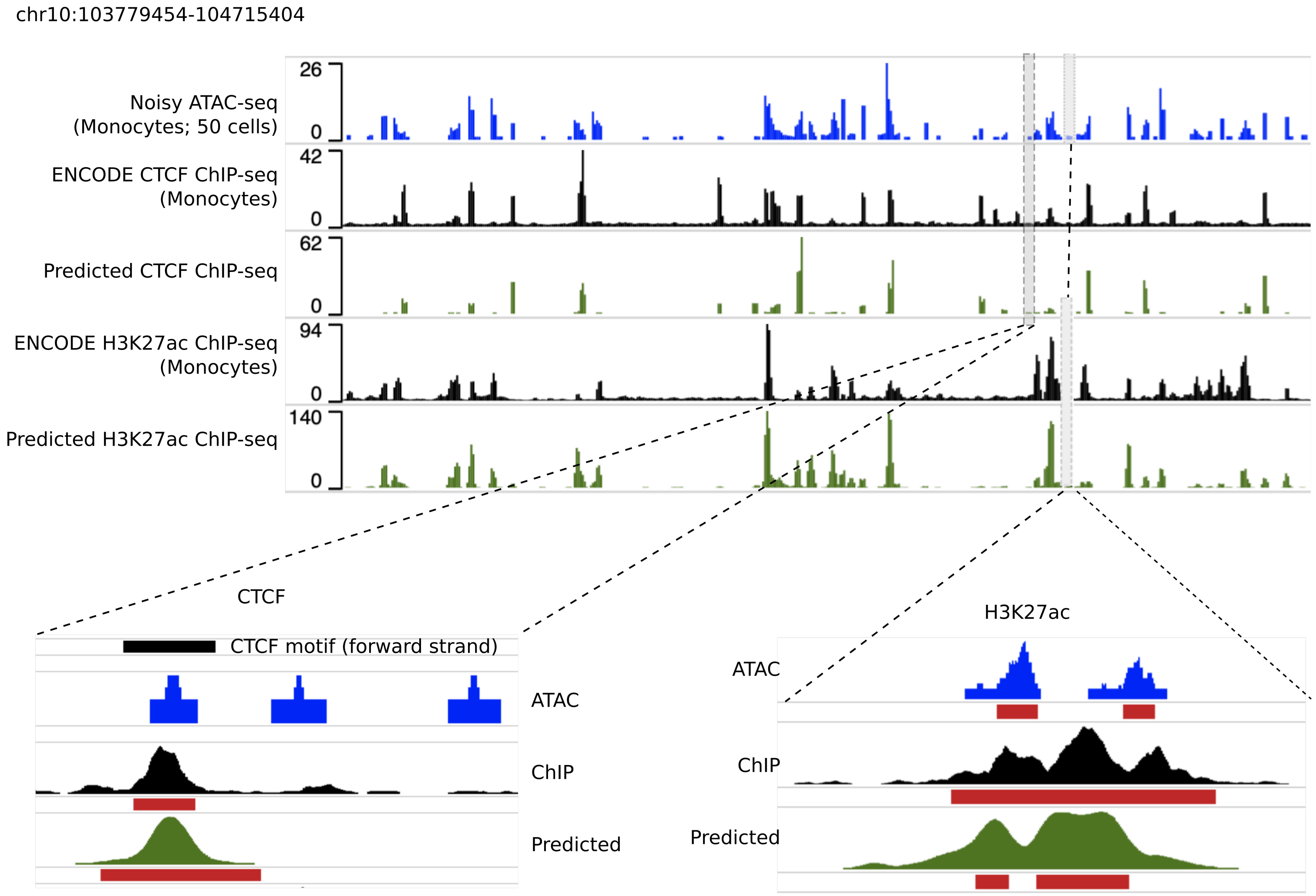

Supplementary Figure 6: Cross-modality prediction of ChIP-seq signal from ATAC-seq. From top to bottom: Noisy aggregate single-cell ATAC-seq (dscATAC) signal from 50 monocytes (blue). ENCODE ChIP-seq signal for CTCF in monocytes (black). CTCF ChIP-seq signal predicted by AtacWorks from the noisy ATAC-seq data (green). ENCODE ChIP-seq signal for H3K27ac in monocytes (black). H3K27ac ChIP-seq signal predicted by AtacWorks from the noisy ATAC-seq data (green). Detailed views of two regions are shown, one for CTCF ChIP-seq and one for H3K27ac ChIP-seq. Red bars below the signal tracks represent peak calls from MACS2 (for noisy ATAC-seq and ENCODE ChIP-seq) or AtacWorks (for predicted ChIP-seq). CTCF: CCCTC-binding factor.

Supplementary Figure 7

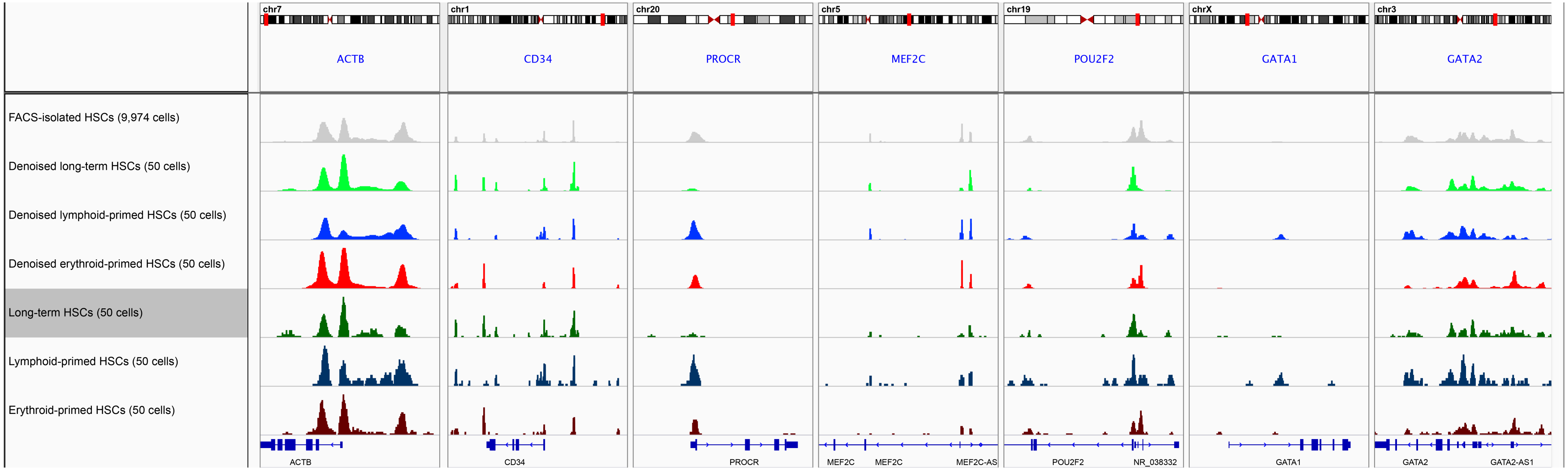

Supplementary Figure 7: AtacWorks denoises dscATAC-seq data from human HSCs. Noisy and denoised ATAC-seq signal tracks for the three aggregated subsamples from Fig. 3. Each subsample was generated by selecting a single HSC and identifying the 50 most similar cells A track for the aggregated FACS-isolated HSCs is also provided for reference. Selected regions contain genes that have been implicated in lineage priming[4, 5]. dscATAC: Droplet Single-Cell ATAC-seq. HSC: hematopoietic stem cell.

Supplementary Figure 8

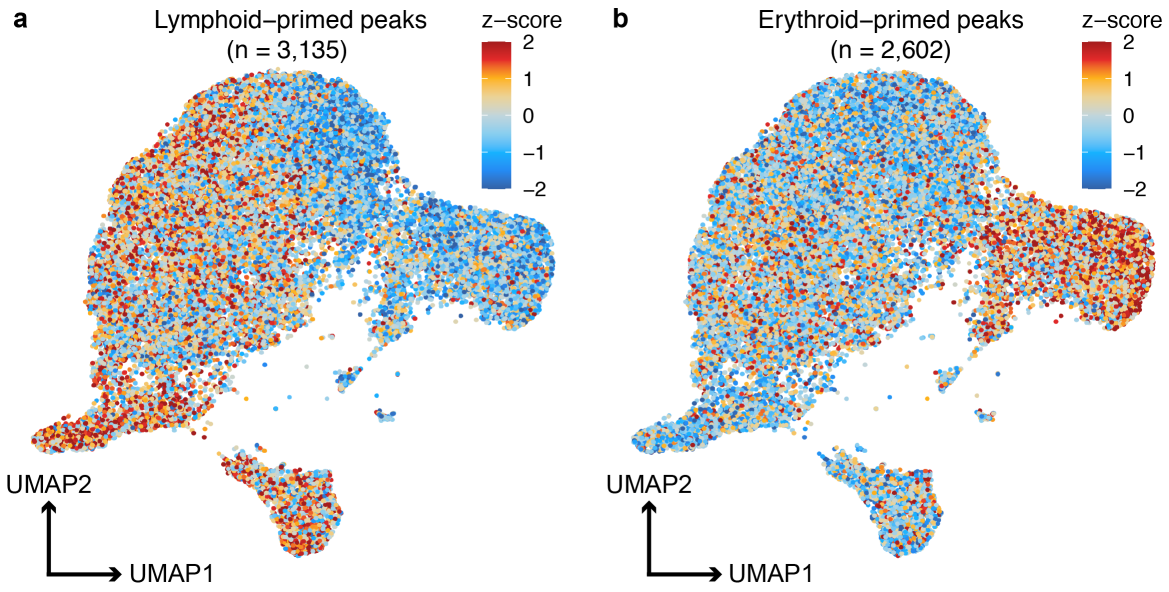

Supplementary Figure 8: Validating the association of regulatory elements with lineage priming. a) A combined UMAP dimensionality reduction of single-cell ATAC-seq profiles from 9,974 HSCs and 28,505 previously-published bead-enriched CD34+ bone marrow progenitor cells[6]. Each cell is colored by a combined chromatin accessibility z-score for 3,135 putative lymphoid-primed peaks identified through permutation analysis and filtering. Compare to Fig. 3b for CD34+ cell type identities. (b) Same as (a), but for 2,606 putative erythroid-primed peaks. HSC: hematopoietic stem cell. Source data are provided as a Source Data file.

Supplementary Figure 9

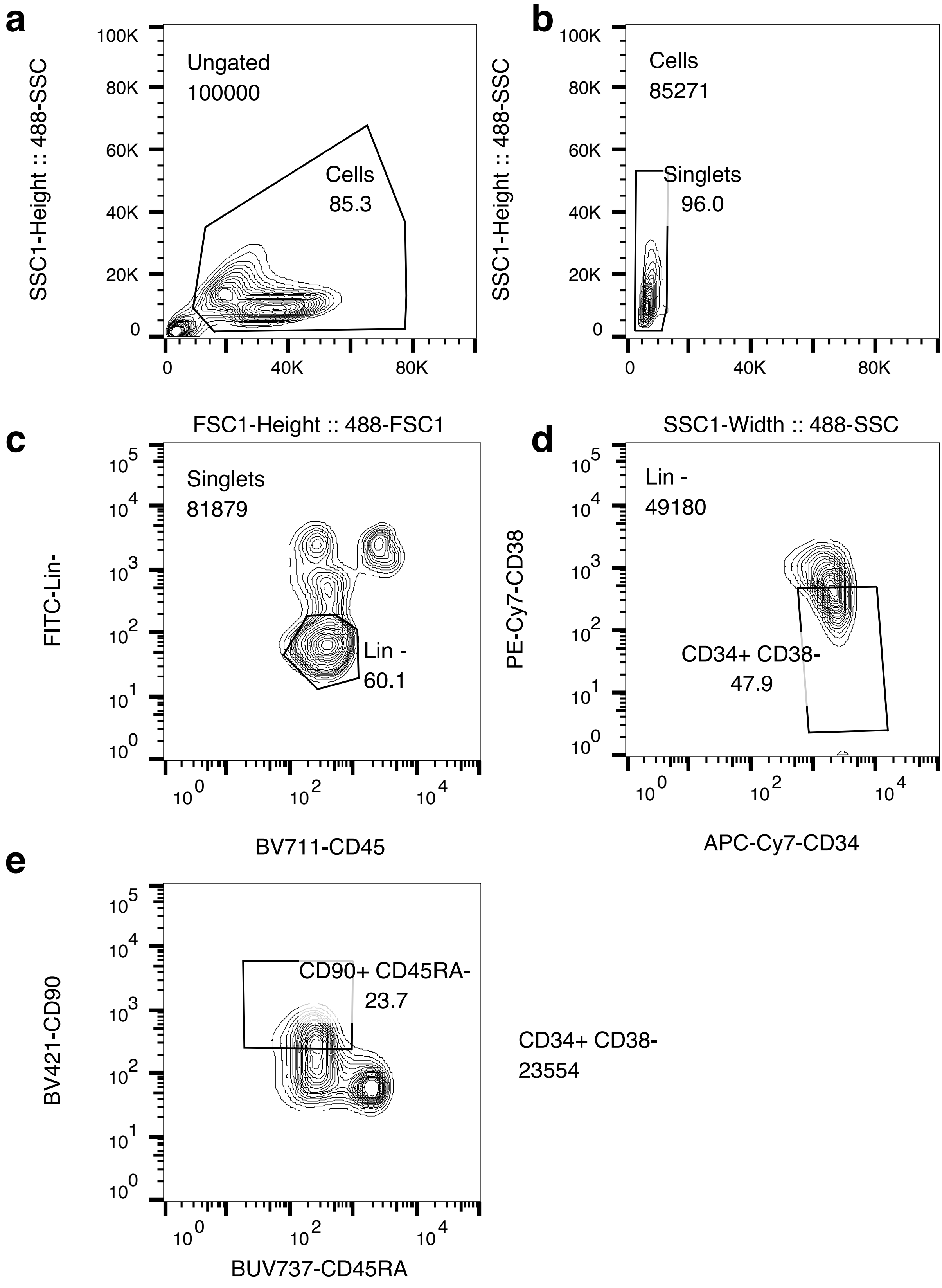

Supplementary Figure 9: Representative example of the sorting strategy used to sort HSCs (CD45+ Lin- CD38- CD34+ CD45RA- CD90+ fraction). The sorting order is shown in the figure (a to e), with the events selected in the gate in plot 1 used in plot 2, events selected in the gate in plot 2 used in plot 3, etc. The analysis was started with 100,000 events and the percentage of events in each gate is shown on the plot, with the total number of events selected in each gate shown in the subsequent plot. The analysis was done using FlowJo v10.7. HSC: hematopoietic stem cell.

Supplementary References

1. Rai, V. *et al.* Single-cell ATAC-Seq in human pancreatic islets and deep learning upscaling of rare cells reveals cell-specific type 2 diabetes regulatory signatures. *Mol Metab* **32**, 109–121 (2020).
2. Koh, P. W., Pierson, E. & Kundaje, A. Denoising genome-wide histone ChIP-seq with convolutional neural networks. *Bioinformatics* **33**, i225–i233 (2017).
3. Schep, A. N., Wu, B., Buenrostro, J. D. & Greenleaf, W. J. chromVAR: inferring transcription-factor-associated accessibility from single-cell epigenomic data. *Nat. Methods* **14**, 975–978 (2017).
4. Buenrostro, J. D. *et al.* Integrated Single-Cell Analysis Maps the Continuous Regulatory Landscape of Human Hematopoietic Differentiation. Cell 173, 1535–1548.e16 (2018).
5. Weinreb, C., Rodriguez-Fraticelli, A., Camargo, F. D. & Klein, A. M. Lineage tracing on transcriptional landscapes links state to fate during differentiation. *Science* **367**, (2020).
6. Lareau, C. A. *et al.* Droplet-based combinatorial indexing for massive-scale single-cell chromatin accessibility. *Nat. Biotechnol.* **37**, 916–924 (2019).
