# Supplementary material for: Deep learning-based enhancement of epigenomics data with AtacWorks
Source: Nat Commun. 2021 Mar 8;12:1507. doi: 10.1038/s41467-021-21765-5 (PMC7940635; doi:10.1038/s41467-021-21765-5)
Supplement: Supplementary file 3 — Description of Additional Supplementary Files [file 41467_2021_21765_MOESM3_ESM.pdf]

Description of Additional Supplementary Files

Supplementary Data 1: Performance of AtacWorks on dscATAC data from mouse brain cells. The performance of AtacWorks was measured on dscATAC data from mouse brain excitatory neurons (EN04 and EN12). The ResNet models used were trained on dscATAC data from human B cells and monocytes. MSE: Mean Squared Error. AUPRC: Area Under the Precision-Recall Curve. AUROC: Area Under the Receiver-Operator Characteristic. dscATAC: Droplet Single-Cell ATAC-seq.

Supplementary Data 2: Cross-modality prediction with AtacWorks. Performance of AtacWorks in predicting CTCF and H3K27ac ChIP-seq from noisy ATAC-seq. ResNet models were trained using aggregate single-cell ATAC-seq (dscATAC) data from B cells and tested on aggregate dscATAC data from monocytes.

Supplementary Data 3: Putative lineage priming regulatory regions. Genomic regulatory regions with significant changes in chromatin accessibility relative to a permuted background in subsamples of lineage-primed hematopoietic stem cells. Significance values were calculated using a two-sided z-test, converted to p-values, and adjusted for multiple comparisons using the Benjamini-Hochberg procedure.
