# Supplementary material for: Deep learning-based enhancement of epigenomics data with AtacWorks
Source: Nat Commun. 2021 Mar 8;12:1507. doi: 10.1038/s41467-021-21765-5 (PMC7940635; doi:10.1038/s41467-021-21765-5)
Supplement: Supplementary file 7 — Reporting Summary [file 41467_2021_21765_MOESM7_ESM.pdf]

nature research

Corresponding author(s): Johnny Israeli, Jason D. Buenrostro

Last updated by author(s): Oct 19, 2020

Reporting Summary

Nature Research wishes to improve the reproducibility of the work that we publish. This form provides structure for consistency and transparency in reporting. For further information on Nature Research policies, see our Editorial Policies and the Editorial Policy Checklist.

Statistics

For all statistical analyses, confirm that the following items are present in the figure legend, table legend, main text, or Methods section.

n/a	Confirmed	
☐	☒	The exact sample size (n) for each experimental group/condition, given as a discrete number and unit of measurement
☐	☒	A statement on whether measurements were taken from distinct samples or whether the same sample was measured repeatedly
☐	☒	The statistical test(s) used AND whether they are one- or two-sided<br>*Only common tests should be described solely by name; describe more complex techniques in the Methods section.*
☐	☒	A description of all covariates tested
☐	☒	A description of any assumptions or corrections, such as tests of normality and adjustment for multiple comparisons
☐	☒	A full description of the statistical parameters including central tendency (e.g. means) or other basic estimates (e.g. regression coefficient) AND variation (e.g. standard deviation) or associated estimates of uncertainty (e.g. confidence intervals)
☒	☐	For null hypothesis testing, the test statistic (e.g. F, t, r) with confidence intervals, effect sizes, degrees of freedom and P value noted<br>*Give P values as exact values whenever suitable.*
☒	☐	For Bayesian analysis, information on the choice of priors and Markov chain Monte Carlo settings
☒	☐	For hierarchical and complex designs, identification of the appropriate level for tests and full reporting of outcomes
☐	☒	Estimates of effect sizes (e.g. Cohen's d, Pearson's r), indicating how they were calculated

Our web collection on statistics for biologists contains articles on many of the points above.

Software and code

Policy information about availability of computer code

Data collection

For flow cytometry, Beckman Coulter MoFlo Astrios EQ Cell Sorter's Summit v62 software was used to collect the data and FCS Express 7 was used to analyze the data.

Data analysis

Per-read bead barcodes were parsed and trimmed using UMI-TOOLs (version 1.0.0, https://github.com/CGATOxford/UMI-tools). Constitutive elements of the bead barcodes were assigned to the closest known sequence allowing for up to 1 mismatch per 6-mer or 7-mer (mean >99% parsing efficiency across experiments).

Paired-end reads were aligned to hg19 using BWA (version 0.7.17, http://bio-bwa.sourceforge.net) on the Illumina BaseSpace online application. Bead-based ATAC-seq processing (BAP) (https://github.com/caleblareau/bap), to identify systematic biases (i.e. reads aligning to an inordinately large number of barcodes), barcode-aware deduplicate reads, and perform merging of multiple bead barcode instances associated with the same cell. Barcode merging is necessary due to the nature of the BioRad SureCell scATAC-seq procedure used in this study, which enables multiple beads per droplet. BAP uses as input an alignment (.bam) file for a given experiment with a bead barcode identifier indicated by a SAM tag.

Aligned reads were combined using samtools merge (version 1.9, http://www.htslib.org)

Subsamples of the data were generated using custom python scripts (available at https://github.com/zchiang/atacworks_analysis).

Tn5 insertion sites were offset by 4/5 bp and smoothed by 200 bp using custom python scripts (available at https://github.com/zchiang/atacworks_analysis).

Genome coverage was calculated using bedtools genomecov (version 2.26.0, https://bedtools.readthedocs.io/en/latest).

Signal tracks were denoised using AtacWorks (https://github.com/clara-genomics/AtacWorks).

Denoised signal tracks were normalized by total coverage using a custom python script (available at https://github.com/zchiang/atacworks_analysis).

bigWigs were generated using the bedGraphToBigWig utility (https://genome.ucsc.edu/goldenpath/help/bigWig.html)

For manuscripts utilizing custom algorithms or software that are central to the research but not yet described in published literature, software must be made available to editors and reviewers. We strongly encourage code deposition in a community repository (e.g. GitHub). See the Nature Research guidelines for submitting code & software for further information.

Data

Policy information about availability of data

All manuscripts must include a data availability statement. This statement should provide the following information, where applicable:

- Accession codes, unique identifiers, or web links for publicly available datasets
- A list of figures that have associated raw data
- A description of any restrictions on data availability

All of the data, trained models, and output signal tracks described in this paper are available for download at https://atacworks-paper.s3.us-east-2.amazonaws.com.

Bulk ATAC-seq datasets of various blood cell types are available from GEO under accession number GSE74912. From these datasets, B cells, NK cells, CD4+ and CD8+ T cells were used for model training, while erythroblasts and monocytes were used for testing. For the transcription factor footprinting model, NK cells, CD8+ T cells, and multipotent progenitor (MPP) cells were used for training, while HSCs were used for testing. The bulk ATAC-seq dataset for Peyer's Patch is available from ENCODE under experiment ENCSR017RQC.

The dscATAC-seq dataset of hematopoietic stem cells generated for this study is available from GEO under accession number GSE147113 (reviewer access code: efqdgsooldqtpmb).

Other dscATAC-seq and dsciATAC-seq datasets are available from GEO under accession number GSE123581. From these datasets, CD4+ T cells, CD8+ T cells, and pre-B cells were used for model training, while monocytes were used for testing. Bead-isolated CD34+ cells were used for the combined UMAP projection. The sciATAC-seq datasets of B cells, monocytes, and macrophages from primary lung tumor are available from GEO under accession number GSE145194. B cells and monocytes were used for model training, while macrophages were used for testing. The scATAC-seq dataset of FACS-isolated peripheral blood mononuclear cells (PBMCs) is available from GEO under accession number GSE96772. These cells were used to infer cell type labels for CD34+ cells in the combined UMAP projection.

CTCF ChIP-seq tracks are available from ENCODE under experiments ENCSR000DLK (HSCs), ENCSR000ATN (B cells) and ENCSR000AUV (monocytes). H3K27ac ChIP-seq tracks are available from ENCODE under experiments ENCSR000AUP (B cells) and ENCSR000ASJ (monocytes).

Field-specific reporting

Please select the one below that is the best fit for your research. If you are not sure, read the appropriate sections before making your selection.

[x] Life sciences   [ ] Behavioural & social sciences   [ ] Ecological, evolutionary & environmental sciences

For a reference copy of the document with all sections, see nature.com/documents/nr-reporting-summary-flat.pdf

Life sciences study design

All studies must disclose on these points even when the disclosure is negative.

Sample size	The sample size of dscATAC-seq data generated for this study was chosen based on the frequency of hematopoietic stem cells within populations of human bone marrow mononuclear cells, the efficiency of FACS, and sequencing cost.
Data exclusions	dscATAC-seq profiles were excluded from downstream analysis if they had less than 1000 unique nuclear fragments, or if less than 50% of fragments did not fall within hematopoiesis regulatory peaks.
Replication	We performed dscATAC-seq on two biological replicates of hematopoietic stem cells and confirmed that replication was successful using standard ATAC-seq QC metrics.
Randomization	No randomization was necessary in our study.
Blinding	No blinding was necessary in our study.

Reporting for specific materials, systems and methods

We require information from authors about some types of materials, experimental systems and methods used in many studies. Here, indicate whether each material, system or method listed is relevant to your study. If you are not sure if a list item applies to your research, read the appropriate section before selecting a response.

Materials & experimental systems

n/a	Involved in the study
☐	☒ Antibodies
☒	☐ Eukaryotic cell lines
☒	☐ Palaeontology and archaeology
☒	☐ Animals and other organisms
☒	☐ Human research participants
☒	☐ Clinical data
☒	☐ Dual use research of concern

Methods

n/a	Involved in the study
☒	☐ ChIP-seq
☐	☒ Flow cytometry
☒	☐ MRI-based neuroimaging

Antibodies

Antibodies used

1) FITC anti-human Lineage Cocktail (CD3, CD14, CD16, CD19, CD20, CD56); BioLegend; Cat. number: 348801; Clone: UCHT1, HCD14, 3G8, HIB19, 2H7, HCD56; Lot number: B269670
2) Brilliant Violet 711 anti-human CD45 Antibody; BioLegend; Cat. number: 304050; Clone: HI30, Lot number: B264419
3) PE/Cy7 anti-human CD38 Antibody; BioLegend; Cat. number: 303516; Clone: HIT2; Lot number: B250184
4) APC/Cyanine7 anti-human CD34 Antibody; BioLegend; Cat. number: 343514; Clone: 581; Lot number: B284482
5) Brilliant Violet 421 anti-human CD90 (Thy1) Antibody; BioLegend; Cat. number: 328122; Clone: 5E10; Lot number: B248580
6) BUV737 Mouse Anti-Human CD45RA; BD Biosciences; Cat. number: 612846; Clone: HI100, Lot number: B270968

Validation

According to BioLegend's website, "each lot of the antibodies (used in this study) is quality control tested by immunofluorescent staining with flow cytometric analysis". The website also provides citations and control FACS plots for each antibody. According to BD Biosciences' website, the BUV737 Mouse Anti-Human CD45RA is tested for flow cytometry applications. The website also provides citations and control FACS plots for this antibody.

Flow Cytometry

Plots

Confirm that:

☐ The axis labels state the marker and fluorochrome used (e.g. CD4-FITC).

☐ The axis scales are clearly visible. Include numbers along axes only for bottom left plot of group (a 'group' is an analysis of identical markers).

☐ All plots are contour plots with outliers or pseudocolor plots.

☐ A numerical value for number of cells or percentage (with statistics) is provided.

Methodology

Sample preparation

Cryopreserved human bone marrow mononuclear cells were purchased from Allcells (catalog number BM, CR, MNC, 10M). Cells were quickly thawed in a 37°C water bath, rinsed with culture medium (RPMI 1640 medium supplemented with 15% FBS) and then treated with 0.2 U/µL DNase I (Thermo Fisher Scientific) in 2 mL of culture medium at room temperature for 15 min. After DNase I treatment, cells were filtered with a 40 µm cell strainer, washed with MACS buffer (1x PBS, 2 mM EDTA and 0.5% BSA), and cell viability and concentration were measured with trypan blue on the TC20 Automated Cell Counter (Bio-Rad). Cell viability was greater than 90% for all samples. CD34 positive cells were then bead enriched using the CD34 MicroBead Kit UltraPure (Miltenyi Biotec, catalog number 130-100-453) following manufacturer's instructions. The enriched population was then simultaneously stained with CD45, Lineage cocktail, CD34, CD38, CD45RA and CD90 antibodies in MACS buffer for 20 min at 4°C. Stained cells were then washed with MACS buffer and the CD45+ Lin- CD38- CD34+ CD45RA- CD90+ fraction (HSCs) was FACS sorted using a MoFlo Astrios EQ Cell Sorter (Beckman Coulter).

Instrument

Beckman Coulter MoFlo Astrios EQ Cell Sorter

Software

We used Beckman Coulter MoFlo Astrios EQ Cell Sorter's Summit v62 software to collect the data and FCS Express 7 to analyze the data.

Cell population abundance

Donor 1's sorted HSC population was 2.4% of the total number of cells that went into the sorter. Donor 2's sorted HSC population was 7.7% of the total number of cells that went into the sorter.

Gating strategy

We used SSC height x FSC height and SSC height x SSC width to remove dead cells and cell doublets, respectively. We then used Lin x CD45 and selected the Lin- CD45+ population, followed by CD38 x CD34 where we selected the CD38- CD34+ population and finally CD90 x CD45RA where we selected the CD90+ CD45RA- population (HSCs). Single stained cells and Fluorescence Minus One stained cells for CD90 were used as controls.

☐ Tick this box to confirm that a figure exemplifying the gating strategy is provided in the Supplementary Information.
